# Multiacquisition Variable-Resonance Image Combination Magnetic Resonance Imaging to Study Detailed Bone Apposition and Fixation of Cementless Knee System Compared to Cemented Total Knee Replacements

**DOI:** 10.1016/j.artd.2022.06.013

**Published:** 2022-08-30

**Authors:** Gina M. Mosich, Hollis G. Potter, Matthew F. Koff, Sara E. Sacher, Mithun Mishu, Geoffrey H. Westrich

**Affiliations:** a Department of Orthopedic Surgery, Adult Reconstruction and Joint Replacement, Hospital for Special Surgery, New York, NY, USA; b Department of Radiology and Imaging, Hospital for Special Surgery, New York, NY, USA

**Keywords:** MAVRIC MRI, Cementless knee, Cemented knee, Fixation, Implant component, Implant design

## Abstract

**Background:**

The ability to utilize magnetic resonance imaging (MRI) to assess bony fixation may allow a better understanding of implant design and longevity. A new cementless total knee arthroplasty (TKA) was introduced, and we hypothesized that this cementless system would show similar fixation compared to a cemented system as assessed by multispectral MRI.

**Methods:**

Multiacquisition variable-resonance image combination selective MRI was performed in 20 patients implanted with a cementless TKA. A matched control group of 20 patients who had a cemented TKA was also evaluated. Each patellar, femoral, and tibial component was graded globally as well as by specific zones. The patella zones were medial, lateral, superior, and inferior. The femoral and tibial components were divided into 4 zones: anteromedial, anterolateral, posteromedial, and posterolateral. Integration grades were performed for each zone as follows: (1) normal, (2) fibrous tissue, (3) fluid interface, (4) osteolysis. A Chi-square test was performed to detect differences in level of integration grades between patients with cemented and those with cementless TKA.

**Results:**

At average 16-month follow-up, the cementless group grading noted 0/80 (0%) vs 2/76 (2.63%) patellar zones with fluid interface, 0/80 (0%) vs 26/80 (32.5%) femoral zones with fibrous tissue, and 10/80 (12.5%) vs 17/80 (21.25%) tibial zones with fibrous tissue. The analysis showed patellar (*P* < .001), femoral (*P* < .001), and tibial (*P* < .001) components had improved fixation and less percentage of fibrous tissue and fluid present in the cementless TKA.

**Conclusions:**

Utilizing metal suppression MRI, a newer cementless knee implant demonstrated excellent biologic fixation and improved fixation compared to the cemented group.

## Introduction

Aseptic component loosening continues to be the leading cause for revision total knee arthroplasty (TKA) [1,2]. With the increased demand for primary TKA, especially in younger patients, component fixation remains a valid concern despite overall excellent survivorship and clinical outcomes of TKA [3].

The cementless technique offers biologic fixation with the potential for better long-term survivorship, especially in younger patients [[4], [5], [6]]. Early generation cementless implants had numerous design flaws resulting in aseptic loosening and poor survivorship compared to cemented knees. More contemporary cementless knee components utilize highly porous surfaces to promote biologic fixation of the prosthesis [[5], [6], [7], [8]]. Proponents of new-generation cementless knee designs cite numerous studies demonstrating no difference in clinical outcomes or long-term survivorship compared to cemented TKA designs [[9], [10], [11], [12], [13], [14], [15], [16], [17], [18], [19], [20]]. Despite the growing number of studies demonstrating excellent outcomes and survivorship of cementless TKAs, there continues to be concerns regarding initial fixation of cementless knee components compared to cemented knee implants that provide immediate fixation. There are also concerns regarding the increased cost of cementless components, which may influence a surgeon’s choice of implant [[21], [22], [23]]. However, many cost-analysis studies have shown no significant difference in overall hospital cost, which may be in part due to the lack of cement and the shorter operating room times with cementless implants [24].

The body of literature supporting excellent midterm and long-term outcomes of new-generation cementless TKA continues to grow. The purpose of this study was to quantify and compare the fixation of uncemented vs cemented TKA components using advanced magnetic resonance imaging (MRI) techniques. We hypothesize that the cementless total knee system will show similar fixation compared to a cemented total knee system as assessed by multispectral, metal suppression MRI.

## Material and methods

The design and conduct of this clinical trial were approved by our institutional review board before patients were included in the study. A nonconsecutive series of 20 patients who underwent primary TKA utilizing a cementless total knee system (Stryker Triathlon Tritanium; Mahwah, NJ) between July 2019 and September 2020 were prospectively enrolled and underwent multiacquisition variable-resonance image combination selective (MAVRIC-SL), metal suppression MRI on the same knee at an average of 16 months (range 11-25 months) postoperatively. The Triathlon Tritanium cementless knee system utilizes a 3D additively manufactured Tritanium (a form of titanium) metal-backed patella and tibial component and a cobalt-chromium-beaded femoral component. There were no formal criteria used for the selection of cementless total knee candidates. Therefore, a matched control group was used in an effort to minimize selection bias. Patients were matched based on age (±5 years), body mass index (BMI, ±5 kg/m^2^), length of follow-up (±4 months), and gender as best as possible. The comparison group of retrospective matched controls included 20 patients who underwent primary TKA utilizing cemented implants (Stryker Triathlon, Smith and Nephew (Memphis, TN) Legion, and Smith and Nephew Journey II). These patients also underwent MAVRIC MRI on the same knee at an average of 16 months (range 7-36 months) postoperatively at our institution. All knees in the cementless group were of Triathlon posterior stabilized (PS) design with asymmetric patellas. Knees in the cemented group included Triathlon PS (n = 6), Triathlon cruciate retaining (CR) (n = 1), Legion PS (n = 9), Legion constrained (n = 2), and Journey II bicruciate stabilized (BCS) (n = 2). All patellas were resurfaced except for 1 in the cemented group. Bone cement utilized was Simplex P (Stryker, Mahwah, NJ) in all cemented knee implants.

All MRI scans were completed at our institution, and surgery was carried out by a single experienced fellowship-trained arthroplasty surgeon. A standard medial parapatellar approach was utilized in all cases. Patients were excluded if they were younger than 18 years or older than 80 years, had a history of claustrophobia with MRI, or had MRI-incompatible aneurysm clips, artificial heart valves, or pacemakers.

All patients underwent our institution’s standard MRI examination of the knee using the clinical TKA imaging protocol including metal artifact reduction sequence and MAVRIC-SL (multiacquisition variable resonance image combination selective) techniques on a 1.5T clinical scanner (General Electric Healthcare, Waukesha, WI). MAVRIC-SL MRI is a specialized acquisition and reconstruction technique that significantly reduces metal artifact.

MRI review was performed by a fellowship-trained musculoskeletal radiologist specializing in MRI with daily clinical experience in interpretation of arthroplasty MRI (who has over 20 years of experience in assessing bony fixation of knee components). Coronal MAVRIC inversion recovery, MAVRIC proton density weighted images, and fast spin echo images were obtained.

Demographics including age, sex, BMI, laterality, femoral component size, tibial component size, patellar size, and level of constraint were captured and compared between the 2 groups. In order to assess bone apposition and fixation of each component, an MRI grading system was developed. Each patellar, femoral, and tibial component was graded globally as well as by specific zones. These zones were obtained by dividing the patellar component into 4 zones (medial, lateral, superior, and inferior). Femoral and tibial components were also divided into 4 zones (anteromedial, anterolateral, posteromedial, and posterolateral). Integration grades were performed for each zone as follows: (1) normal, (2) fibrous tissue, (3) fluid interface, (4) osteolysis. To quantify integration, the percentage of integration was measured in thirds for each component zone, 0%-33% integration, 33%-66% integration, and greater than 66% integration.

A Chi-square test was performed to detect differences in distribution of sex, laterality, and level of integration grade between patients with cemented and those with cementless TKA. A nonparametric t-test was performed to detect difference in age by fixation technique. Statistical analyses were performed with MATLAB Version 2020A (MathWorks, Inc., Natick, MA).

## Results

Demographic data between the 2 groups are shown in Table 1. The uncemented group had 8 men and 12 women. The cemented group had 5 men and 15 women. No difference in patient age, laterality, or sex was found between the 2 groups (Table 1)

**Table 1:** Demographics.

Demographics	Triathlon cementless	Cemented	*P* values
Age	63.8 ± 4.54	64.2 ± 8.38	.163
Gender			.999
Male	8	5	
Female	12	15	
BMI	30.63 ± 6.28 kg/m^2^	29.71 ± 6.96 kg/m^2^	.663
Mean F/U	16 ± 4.38 mo	16 ± 6.62 mo	.999
Laterality			.999
Right	10	12	
Left	10	8	

There were no significant differences in global bony integration in the patellar, femoral, and tibial components between the cementless and the cemented knee systems. Zonal analysis showed there was no osteolysis in any knee system regardless of fixation. Of note, 1 patient in the cemented cohort did not have their patella resurfaced, so there were only 76 patella zones to evaluate in the cemented knee cohort. The cementless knee group had 0/80 (0%) patellar zones graded as fluid interface present, while the cemented group had 2/76 (2.63%) patellar zones graded as fluid interface present, *P* <.001. In the cementless group, 78/80 (97.5%) patellar zones demonstrated >66% integration, whereas only 66/76 (86.8%) of patellar zones in the cemented group demonstrated >66% integration (Table 2).

**Table 2:** Patella zonal analysis.

Patellar zone	Integration grade (cementless n = 20)	Integration grade (cemented n = 19)	*P* value
Medial	Normal: 14/20	Normal: 06/19	.95
	Fibrous tissue: 06/20	Fibrous tissue: 12/19	
	Fluid interface: 00/20	Fluid interface: 01/19	
	Osteolysis: 00/20	Osteolysis: 00/19	
Lateral	Normal: 17/20	Normal: 08/19	.99
	Fibrous tissue: 03/20	Fibrous tissue: 10/19	
	Fluid interface: 00/20	Fluid interface: 01/19	
	Osteolysis: 00/20	Osteolysis: 00/19	
Superior	Normal: 18/20	Normal: 13/19	.99
	Fibrous tissue: 02/20	Fibrous tissue: 06/19	
	Fluid interface: 00/20	Fluid interface: 00/19	
	Osteolysis: 00/20	Osteolysis: 00/19	
Inferior	Normal: 18/20	Normal: 14/19	.99
	Fibrous tissue: 02/20	Fibrous tissue: 05/19	
	Fluid interface: 00/20	Fluid interface: 00/19	
	Osteolysis: 00/20	Osteolysis: 00/19	
All zones	Normal: 67/80	Normal: 41/76	<.001
	Fibrous tissue: 13/80	Fibrous tissue: 33/76	
	Fluid interface: 00/80	Fluid interface: 02/76	
	Osteolysis: 00/80	Osteolysis: 00/76	

These zones were obtained by dividing the patellar component into 4 zones (medial, lateral, superior, and inferior). Integration grades were performed for each zone as follows: (1) normal, (2) fibrous tissue, (3) fluid interface, (4) osteolysis.

Regarding femoral components, 0/80 (0%) femoral zones were graded as fibrous tissue present in the cementless group, whereas 26/80 (32.5%) femoral zones graded as fibrous tissue present in the cemented group, *P* < .001. In the cementless group, 80/80 (100%) of femoral zones demonstrated >66% integration, whereas only 70/80 (87.5%) of femoral zones in the cemented group demonstrated >66% integration (Table 3).

**Table 3:** Femoral zonal analysis.

Femoral zone	Integration grade (cementless n = 20)	Integration grade (cemented n = 20)	*P* value
Anteromedial	Normal: 20/20	Normal: 15/20	.99
	Fibrous tissue: 00/20	Fibrous tissue: 05/20	
	Fluid interface: 00/20	Fluid interface: 00/20	
	Osteolysis: 00/20	Osteolysis: 00/20	
Anterolateral	Normal: 20/20	Normal: 19/20	.99
	Fibrous tissue: 00/20	Fibrous tissue: 01/20	
	Fluid interface: 00/20	Fluid interface: 00/20	
	Osteolysis: 00/20	Osteolysis: 00/20	
Posteromedial	Normal: 20/20	Normal: 08/20	.72
	Fibrous tissue: 00/20	Fibrous tissue: 11/20	
	Fluid interface: 00/20	Fluid interface: 01/20	
	Osteolysis: 00/20	Osteolysis: 00/20	
Posterolateral	Normal: 20/20	Normal: 11/20	.97
	Fibrous tissue: 00/20	Fibrous tissue: 09/20	
	Fluid interface: 00/20	Fluid interface: 00/20	
	Osteolysis: 00/20	Osteolysis: 00/20	
All zones	Normal: 80/80	Normal: 53/80	<.001
	Fibrous tissue: 00/80	Fibrous tissue: 26/80	
	Fluid interface: 00/80	Fluid interface: 01/80	
	Osteolysis: 00/80	Osteolysis: 00/80	

Femoral components were divided into 2 zones (anterior and posterior), then each zone was divided in 2 subzones (medial and lateral). Integration grades were performed for each zone as follows: (1) normal, (2) fibrous tissue, (3) fluid interface, (4) osteolysis.

In evaluating the tibial components, 10/80 (12.5%) tibia zones were graded as fibrous tissue present in the cementless group, whereas 17/80 (21.25%) tibial zones were graded as fibrous tissue present in the cemented group, which showed no significant difference between the cementless and cemented group. In the cementless group, 78/80 (97.5%) of tibial zones demonstrated >66% integration, and similarly, 77/80 (96.3%) of tibial zones in the cemented group demonstrated >66% integration (Table 4).

**Table 4:** Tibial zonal analysis.

Tibial zone	Integration grade (cementless n = 20)	Integration grade (cemented n = 20)	*P* value
Anteromedial	Normal: 19/20	Normal: 17/20	.99
	Fibrous tissue: 01/20	Fibrous tissue: 03/20	
	Fluid interface: 00/20	Fluid interface: 00/20	
	Osteolysis: 00/20	Osteolysis: 00/20	
Anterolateral	Normal: 18/20	Normal: 16/20	.99
	Fibrous tissue: 02/20	Fibrous tissue: 04/20	
	Fluid interface: 00/20	Fluid interface: 00/20	
	Osteolysis: 00/20	Osteolysis: 00/20	
Posteromedial	Normal: 17/20	Normal: 16/20	.99
	Fibrous tissue: 03/20	Fibrous tissue: 04/20	
	Fluid interface: 00/20	Fluid interface: 00/20	
	Osteolysis: 00/20	Osteolysis: 00/20	
Posterolateral	Normal: 16/20	Normal: 14/20	.99
	Fibrous tissue: 04/20	Fibrous tissue: 06/20	
	Fluid interface: 00/20	Fluid interface: 00/20	
	Osteolysis: 00/20	Osteolysis: 00/20	
All zones	Normal: 70/80	Normal: 63/80	<.001
	Fibrous tissue: 10/80	Fibrous tissue: 17/80	
	Fluid interface: 00/80	Fluid interface: 00/80	
	Osteolysis: 00/80	Osteolysis: 00/80	

Tibial components were divided in 2 zones (anterior and posterior), then each zone was divided in 2 subzones (medial and lateral). Integration grades were performed for each zone as follows: (1) normal, (2) fibrous tissue, (3) fluid interface, (4) osteolysis.

## Discussion

Cemented fixation continues to be the favored method of fixation for TKA used by the majority of arthroplasty surgeons, as it demonstrates excellent survivorship and clinical function. However, aseptic loosening continues to be the leading cause of revision for TKA, accounting for 31%-39% of revision cases [1,2]. Cement fixation provides robust initial fixation; however, it is subject to tensile and shear forces, which are not well tolerated and can lead to micromotion and component loosening [25,26]. Cementless fixation provides the opportunity for biologic ingrowth with the potential for long-term remodeling and eliminates the risk of cement particle third-body debris [25,26]. This biologic fixation may lower the incidence of aseptic loosening and could provide a superior option for patients most at risk of loosening, namely young and obese patients [27,28].

Numerous studies have demonstrated excellent outcomes and survivorship of new-generation cementless total knees that utilize highly porous surfaces and promote biologic fixation [[15], [16], [17], [18], [19], [20]]. The use of MAVRIC MRI to assess fixation of cementless and cemented TKA is a novel technique used in this study. MAVRIC MRI has been shown to significantly reduce metal artifact and is an established reliable method in the assessment of osteolysis and osseointegration [29,30]. A similar technique of assessing fixation utilizing MAVRIC MRI has been used in symptomatic cemented unicompartmental knee arthroplasty [31]. Our study demonstrates robust fixation of cementless TKA with results that are superior to cemented fixation as demonstrated on metal suppression MRI at, on average, 16 months postoperatively. The cementless knee group had 0/80 (0%) patellar zones with fluid interface present, while the cemented group had 2/76 (2.63%) patellar zones with fluid interface present. In the cementless knees, 0/80 (0%) femoral zones demonstrated presence of fibrous tissue, whereas in the cemented group, 11/80 (14.47%) femoral zones had fibrous tissue present. While these findings likely represent no clinically significant difference with regard to overall fixation of the knee systems as none of the knees in either group demonstrated osteolysis or loosening, this does raise concern for long-term fixation. However, these results are reassuring that the early fixation of cementless total knee components are comparable, if not superior, to cemented total knees. Further study is required with larger patient numbers and longer term follow-up to better assess fixation between cementless and cemented knees.

This study has several limitations. First, this study includes a relatively small number of patients, and results may vary when looking at larger populations. This was not a consecutive series, and there may be selection bias between the patients chosen for cementless vs cemented fixation based on age, perceived bone quality, or other factors. No formal selection criteria were used in this study for cementless fixation, and therefore, a matched control group based on age, gender, and BMI was used to minimize selection bias. Second, all surgeries were performed at a single institution, and results may not be generalizable to other populations. Also, this study looked at 1 cementless implant design, and findings may not be generalizable to other cementless implant designs. Studies have shown that not all cementless knees are designed equally, with certain design features such as highly porous coating, pegs, and longer keel resulting in better fixation [32]. The cementless knee implant evaluated in this study has demonstrated excellent functional outcomes compared to its cemented counterpart in studies with midterm follow-up [20]. It is notable that different manufacturers and different knee design types were compared, which may influence result findings. Finally, the duration of follow-up in this study was relatively short; however, biologic fixation was achieved in 100% of the cementless knees evaluated for the femur, tibia, and patellar components. Further studies evaluating the fixation of cementless TKA for long term are necessary. However, given the concern for early fixation of cementless TKA, evaluation of the fixation interface at 16 months postoperatively provides valuable information in the decision to use cementless design implants.

## Conclusions

This study demonstrates improved fixation with lower percentage of fibrous tissue and fluid in the cementless knee system than those in a control group of cemented knees at 16 months of follow-up. In our single-surgeon study, the cementless knee system employed demonstrated excellent biologic fixation.

## Funding

Research support received from 10.13039/100008894Stryker Corporation.

## Conflicts of interest

Dr. Westrich receives royalties and research support as a principal investigator and is a paid consultant for Stryker; is in the speakers' bureau of or gave paid presentations for Ethicon; and is a board member at the Eastern Orthopedic Association. Dr. Koff is in the editorial review board of the Journal of Orthopaedic Research. All other authors declare no potential conflicts of interest.

For full disclosure statements, refer to https://doi.org/10.1016/j.artd.2022.06.013.

## Appendix A Supplementary Data

Conflict of Interest Statement for Westrich

Conflict of Interest Statement for Potter

Conflict of Interest Statement for Koff

Conflict of Interest Statement for Mishu

Conflict of Interest Statement for Mosich

Conflict of Interest Statement for Sacher
